# Initiation and propagation kinetics of inhibited lipid peroxidation

**DOI:** 10.1038/s41598-021-86341-9

**Published:** 2021-03-25

**Authors:** Reza Farhoosh

**Affiliations:** Department of Food Science and Technology, Ferdowsi University of Mashhad, Faculty of Agriculture, P.O. Box: 91775-1163, Mashhad, Iran

**Keywords:** Plant sciences, Chemistry

## Abstract

Effect of hydroxytyrosol (HT) and *tert*-butylhydroquinone (TBHQ) on the kinetics of lipid hydroperoxides (LOOH) accumulation during the initiation and propagation peroxidations of canola and fish oils at 60 °C was studied. The initiation kinetics of the inhibited peroxidation indicated considerable relative activities, *A*, for HT and TBHQ in the canola (> 3200 and > 27,000, respectively) and fish (> 120 and > 5000, respectively) oils. The critical concentrations of LOOH reverse micelles (CMC_L_ = 33 mM and 57 mM in the canola and fish, respectively, oils) significantly decreased, on average, to about one-third and 8% of the initial values for HT and TBHQ, respectively. Interestingly, the propagation kinetics of the inhibited peroxidation demonstrated that the antioxidants were still able to inhibit peroxidation, so that the relative propagation oxidizability parameter *R*_n_′ was significantly improved to < 0.5 for HT and to < 0.2 for TBHQ in the canola and fish, respectively, oils.

## Introduction

Lipid hydroperoxides (LOOH) as the primary products of lipid peroxidation turn readily to a wide variety of secondary decomposition products of undesirable impacts on the sensory attributes and health of lipid systems^1^. Any strategies employed to prevent peroxidation or to evaluate the extent of the reaction should take into account both primary and secondary products^2^. However, secondary oxidation products would not tangibly be produced, especially at mild temperatures (≤ 60 °C)^2^, below a critical LOOH concentration, [LOOH]_IP_^3^.

**Figure 1:**
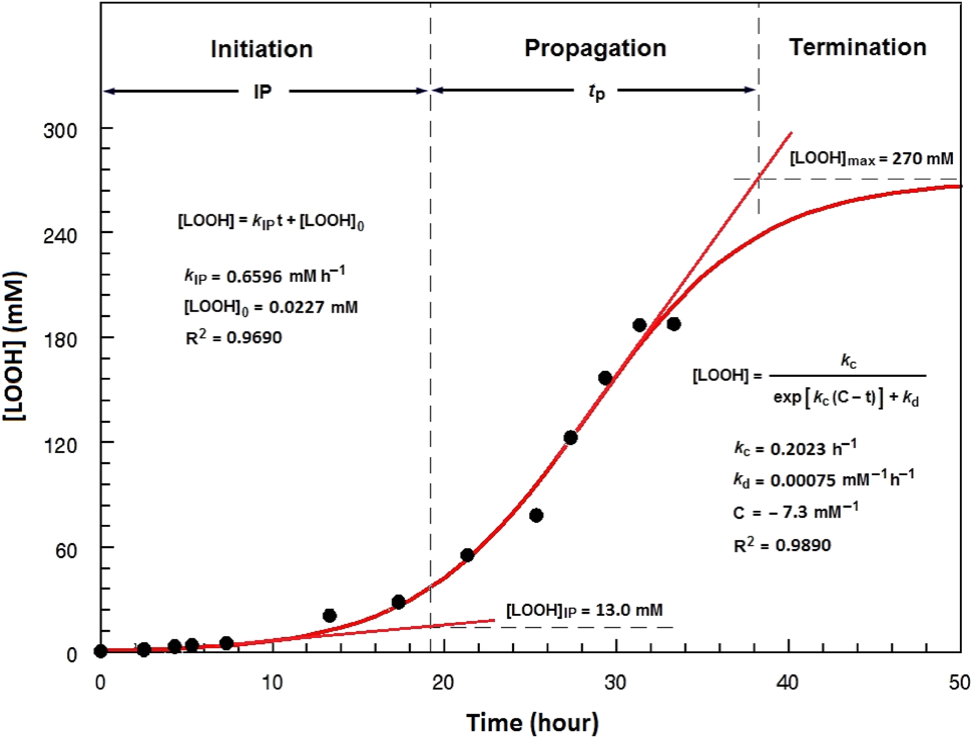
Kinetic curve of the accumulation of lipid hydroperoxides (LOOH) during peroxidation of the fish oil containing 1.2 mM of hydroxytyrosol at 60 °C, and the kinetic data from the linear and sigmoidal functions fitted on the initiation phase and the whole range, respectively, of the data points. *IP* induction period, *t*_*p*_ duration of the propagation phase, *[LOOH]*_*0*_ LOOH concentration at *t* = 0, *[LOOH]*_*IP*_ LOOH concentration at IP, *[LOOH]*_*max*_ the maximum concentration of LOOH, *k*_*IP*_ the pseudo-zero order rate constant of the initiation phase, *k*_*c*_ a composite (pseudo-first order) rate constant, *k*_*d*_ the decomposition (pseudo-second order) rate constant of the bimolecular reaction of preformed LOOH, *C* an integration constant.

During the initiation phase of lipid peroxidation, which is known as induction period (IP), [LOOH] increase very slowly and reach the [LOOH]_IP_ (Fig. 1). The reaction is of a pseudo-zero kinetic order with the rate constant *k*_IP_^4^. Afterwards, [LOOH] steeply increase over a time range called as the propagation phase (*t*_p_) and asymptotically reach the maximum value [LOOH]_max_ during the termination phase. The whole kinetic time range has well described by a sigmoidal function providing two kinetic rate constants *k*_c_ and *k*_d_ of pseudo-first and -second kinetic orders, respectively^4,5^.

Antioxidant addition has always been as one of the efficient strategies to inhibit lipid peroxidation. The LOOH-based evaluations of antioxidants activity have basically been relying only upon the kinetics of antioxidant molecules during the initiation phase of lipid peroxidation. On this basis, the antioxidants of higher strength are those causing more decreases in the value of *k*_IP_ whereas more effective ones provide higher values of IP. The common belief is that IP continues until the antioxidant has been destroyed and its duration is proportional to the antioxidant concentration. The peroxidation after IP, therefore, continues at a rate equal to that of unprotected peroxidations^6^. This is against our initial findings, so that the kinetic parameters and rate constants characterizing the propagation phase might be of considerable importance to evaluate the activity of antioxidants.

**Figure 2:**
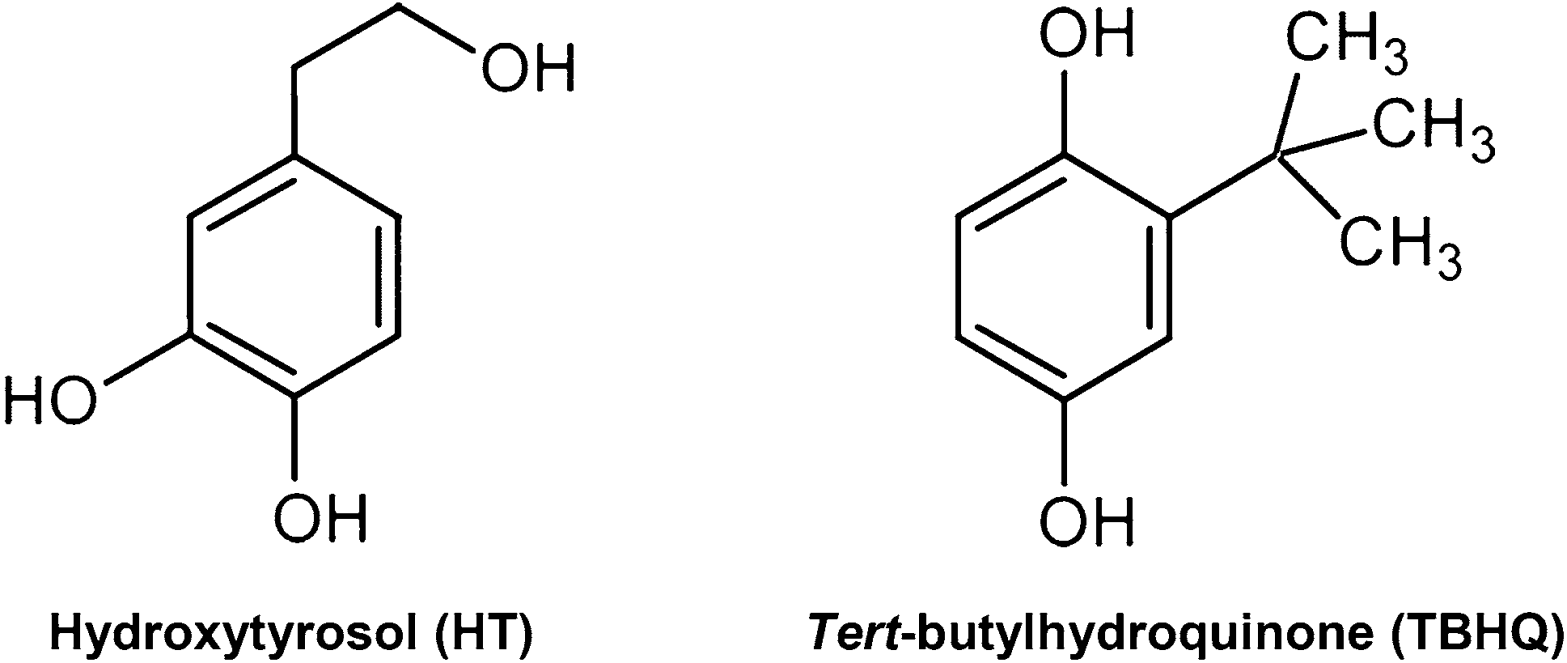
Molecular structure of hydroxytyrosol and *tert*-butylhydroquinone.

Considering the methodology recently developed to analyze the oxidative stability of bulk lipid systems^5^, the present study aimed to concomitantly investigate the initiation and propagation kinetics of the inhibited (hydroxytyrosol, HT, and *tert*-butylhydroquinone, TBHQ; Fig. 2) peroxidation of the stripped canola and fish oils of quite different fatty acid compositions. HT (3,4-dihydroxyphenylethanol) is one of the major compounds of the phenolic fraction of olive fruits and can be found in virgin olive oil and the wastes generated during olive oil processing. In vitro evaluation of antioxidant and biological properties of HT has been of great interest in recent years. It has been found to possess antioxidant activity higher than antioxidant vitamins and many of synthetic antioxidants^7^. TBHQ is a diphenolic synthetic antioxidant of very high effectiveness which is broadly used in edible fats and oils^8^.

## Materials and methods

### Materials

Commercial canola oil was purchased from a local market. Kilka fish oil was provided by Khazar Company (Babolsar, Mazandaran, Iran). The oil samples were stored at − 18 °C until analysis. HT (powder, purity ≥ 98%) (PubChem CID: 82755) was supplied by Hunan World Well-Being Bio-Tech (China, Mainland). Other chemicals and solvents used were of analytical reagent grade and purchased from Merck (Darmstadt, Germany) and Sigma-Aldrich (St. Louis, MO).

### Purification and preparation of the oils

The oil samples were purified twice by adsorption column chromatography. Glass columns (40 × 3.5 cm i.d.) for the canola oil were packed by aluminum oxide 60 (active, neutral, 120 g) activated at 240 °C for 4 h right before use. As for the fish oil sample, the activated aluminum oxide 60 (55 g) was the bottom layer, and the middle and top layers were silica gel (60–200 mesh, 80 g) activated at 160 °C for 3 h right before use and activated carbon (2 g), respectively. The chromatographic columns and collection vessels were wrapped in aluminum foil, and the oils were drawn through the column by suction without solvent^9^. To make sure complete purification of the oils, the contents of total phenolics, total tocopherols, and hydroperoxides (see below) were determined in the purified oils. The oils containing HT (0.6 and 1.2 mM) and TBHQ (0.6 mM) were prepared by adding aliquots of their solutions in acetone. The acetone was removed under a steam of nitrogen.

### Total phenolics

The oil (2.5 g) was dissolved in 2.5 ml of *n*-hexane and extracted three times by 5-min centrifugations (2700×*g*) with CH_3_OH:H_2_O (80:20 v/v). After adding 2.5 ml of Folin–Ciocalteau reagent and 5 ml of 7.5% Na_2_CO_3_ to the extract, the solution was made up to 50 ml with distilled water. The solutions were stored overnight and the spectrophotometric analysis was performed at 765 nm. To plot calibration curve, 1 ml of methanolic solutions of gallic acid (0.04–0.40 mg/ml), 6 ml of methanol, 2.5 ml of the Folin–Ciocalteau reagent, and 5 ml of 7.5% Na_2_CO_3_ were made up to 50 ml with distilled water. TP was reported as milligrams of gallic acid per kilogram of oil^10^.

### Total tocopherols

A solution of 100 ± 10 mg of the oil in 5 ml of toluene was prepared. After successive addition of 3.5 of 2,2′-bipyridine (0.07% w/v in 95% aqueous ethanol) and 0.5 ml of FeCl_3_.6H_2_O (0.2% w/v in 95% aqueous ethanol), the solution was made up to 10 ml with 95% aqueous ethanol. After 1 min, the absorbance was read at 520 nm against a blank containing all the reagents except the oil. All the operations were carried out under subdued light. A calibration curve of α-tocopherol in toluene (0–240 µg/ml) was prepared. TT was reported as milligrams of α-tocopherol per kilogram of oil^11^.

### Partition coefficient (log P)

Solutions (0.3 mM) of each antioxidant in 1-octanol was heated at 60 °C for 1 h to solubilize antioxidants. The maximum absorbance was read by UV spectrum (A_0_). Equal volumes of this solution and acetate buffer (0.1 M, pH 5.5) were vortexed (70×*g*) for 1 min. The UV spectrum of the 1-octanol layer was determined after 30 min (Ax). The partition coefficient (log P) of antioxidant was calculated according to Eq. (1)^12^:

1 $$ {\text{log P = log }}\left( {\frac{{{\text{A}}_{{\text{x}}} }}{{{\text{A}}_{{0}} - {\text{A}}_{{\text{x}}} }}} \right) $$

### Fatty acid composition

Fatty acids were transesterified into methyl esters by vigorous shaking of a solution of oil in *n*-hexane (0.3 g in 7 ml) with 2 ml of 7 N methanolic potassium hydroxide at 50 °C for 10 min. The methyl esters were identified using an HP-5890 gas chromatograph (Hewlett-Packard, CA, USA) equipped with a CP-FIL 88 (Supel Co., Inc., Bellefonte, PA) capillary column of fused silica, 60 m in length × 0.22 mm I.D., 0.2 µm film thickness, and a flame ionization detector. Nitrogen was used as carrier gas with a flow rate of 0.75 ml min^−1^. The temperature of oven, injector and detector was maintained at 198, 250 and 250 °C, respectively. The fatty acid compositions were reported in relative area percentages with the average of duplicate samples^13^.

### Peroxidation

The concentration (mM) of LOOH (known as peroxide value, PV; see below) was determined over time in a kinetic regime^14^ in which the reaction medium is saturated with oxygen through performing the process in layers of a thickness less than 1 mm. In such a condition, more reproducible kinetic parameters are achieved and the rate of LOOH accumulation is independent of oxygen concentration^15^. The 1-mm layers of the oils (4 g) in Petri dishes of 9 cm in diameter were stored in a dry oven set at 60 °C.

### PV measurement

The PV of the oil samples was measured spectrophotometrically at 500 nm by a UV–Vis instrument (Model 160A Shimadzu, Kyoto, Japan). The oil samples were mixed in with 9.8 ml chloroform–methanol (7:3 v/v) on a vortex mixer for 2–4 s. Ammonium thiocyanate solution (50 ml, 30% w/v) and 50 ml of iron (II) chloride solution ([0.4 g barium chloride dihydrate dissolved in 50 ml H_2_O] + [0.5 g FeSO_4_.7H_2_O dissolved in 50 ml H_2_O] + 2 ml 10 M HCl, with the precipitate, barium sulfate, filtered off to produce a clear solution]) were added, respectively, and after adding each of them, the sample was mixed on a vortex mixer for 2–4 s. Then, the absorbance of the sample was read, after 5 min incubation at room temperature^16^. Results in milliequivalents of oxygen per kilogram of oil were reported as LOOH molarity (1 meq kg^–1^ = 0.504 mM)^15^.

### Kinetic parameters derived from the LOOH accumulation curves

The method developed recently by the author was employed to calculate the LOOH-based kinetic parameters^4,5^. The concentration of LOOH (mM) linearly increased during IP (Fig. 1) according to

2 $$ {\text{[LOOH]}} = k_{{{\text{IP}}}} {(}t{)} + {\text{[LOOH]}}_{{0}} , $$

where *k*_IP_ (mM h^–1^) and [LOOH] at *t* = 0 are the equation parameters. The increase pattern of [LOOH] over the whole kinetic time range was described by

3 $$ {\text{[LOOH] = }}\frac{{k_{{\text{c}}} }}{{\exp \left[ {k_{{\text{c}}} \left( {{\text{C}} - t} \right)} \right] + k_{{\text{d}}} }}, $$

where *k*_c_ (h^−1^) represents propagation oxidizability; *k*_d_ (mM^−1^ h^−1^) stands for the kinetic rate constant of the bimolecular decomposition of the LOOH produced in the propagation phase; and C (mM^−1^) is an integration constant. The value [LOOH]_max_ (mM) was calculated from

4 $$ {\text{[LOOH]}}_{{{\text{max}}}} {\text{ = lim}}_{t \to \infty } \left\{ {\frac{{k_{{\text{c}}} }}{{\exp \left[ {k_{{\text{c}}} \left( {{\text{C}} - t} \right)} \right] + k_{{\text{d}}} }}} \right\} = \frac{{k_{{\text{c}}} }}{{k_{{\text{d}}} }}. $$

The maximum rate of LOOH production (*R*_max_, mM h^−1^) and the propagation oxidizability parameter (*R*_n_, h^−1^) were calculated from Eqs. (5) and (6), respectively.

5 $$ R_{{{\text{max}}}} = \left( {\frac{{d{\text{[LOOH]}}}}{dt}} \right)_{{{\text{max}}}} = \frac{{k_{{\text{c}}}^{{2}} }}{{4k_{{\text{d}}} }}, $$

6 $$ R_{{\text{n}}} { = }\frac{{R_{{{\text{max}}}} }}{{[{\text{LOOH}}]_{{{\text{max}}}} }}. $$

Equations (7) and (8) gave IP (h) and [LOOH]_IP_ (mM):

7 $$ {\text{IP}} = \frac{{k_{{\text{c}}} \left( {2 - k_{{\text{c}}} {\text{C}} + \ln k_{{\text{d}}} } \right) - 4{\text{[LOOH}}]_{{0}} k_{{\text{d}}} }}{{4k_{{{\text{IP}}}} k_{{\text{d}}} - k_{{\text{c}}}^{{2}} }}, $$

8 $$ [{\text{LOOH}}]_{{{\text{IP}}}} = k_{{{\text{IP}}}} {\text{(IP)}} + {\text{[LOOH]}}_{{0}} . $$

The initiation oxidizability parameter *O*_i_ (mM^−1^ h^2^), representing lipid oxidizability only with respect to the initiation phase, and *t*_p_ (h) were calculated as follow:

9 $$ O_{{\text{i}}} { = }\frac{{{\text{IP}}}}{{k_{{{\text{IP}}}} }}, $$

10 $$ t_{{\text{P}}} = \frac{{4k_{{\text{d}}} R_{\max } - k_{{\text{c}}} R_{n} \left( {2 - k_{{\text{c}}} {\text{C}} + \ln k_{{\text{d}}} } \right)}}{{4k_{{\text{d}}} R_{\max } R_{n} }} - {\text{IP}}{.} $$

### Initiation kinetics of inhibited peroxidation

According to the terminology introduced by Yanishlieva and Marinova^6^, the effectiveness of an antioxidant, which means its capability to scavenge peroxyl radicals (LOO^**∙**^), is calculated by the stabilization factor *F*:

11 $$ F{ = }\frac{{{\text{IP}}_{{{\text{AH}}}} }}{{{\text{IP}}_{{\text{C}}} }}, $$

where IP_AH_ and IP_C_ are the IPs in the presence and absence (control) of the antioxidants (AH), respectively. Oxidation rate ratio (ORR) as an inverse measure of antioxidant strength was generated by Eq. (12):

12 $$ {\text{ORR = }}\frac{{k_{{\text{IP, AH}}} }}{{k_{{\text{IP, C}}} }}, $$

where *k*_IP,AH_ and *k*_IP,C_ are the values of *k*_IP_ in the presence and absence (control) of AH, respectively. Antioxidant activity was calculated by the unifying parameter *A*:

13 $$ A{ = }\frac{F}{{{\text{ORR}}}}{ = }\frac{{O_{{\text{i, AH}}} }}{{O_{{\text{i, C}}} }}. $$

### Statistical analysis

All determinations were carried out in triplicate and data were subjected to analysis of variance (ANOVA). ANOVA and regression analyses were carried out by the MStatC and Slide Write 7.0. Significant differences were determined by Duncan’s multiple range tests. P values < 0.05 were considered statistically significant.

## Results and discussion

### Chemical composition of the oil samples

The chromatographic technique yielded the stripped oils containing no detectable LOOH, tocopherols, and phenolic compounds. The canola and Kilka fish oils possessed the same fatty acid compositions as the corresponding ones usually reported in literature (Table 1). Due to the content of mainly palmitic acid (C16:0), the fish oil had significantly higher saturation degree (SFA) than the canola oil. The canola oil was constituted of more remarkable content of monounsaturated fatty acids (MUFA, mainly oleic acid, C18:1) compared to the fish oil (mainly palmitoleic, C16:1, and oleic acids). While linoleic (C18:2) and linolenic (18:3) acids were the main types of polyunsaturated fatty acids (PUFA) measured in the canola oil, the majority of PUFA found in the fish oil were linoleic acid as well as the highly oxidizable eicosapentaenoic (EPA, C20:5) and docosahexaenoic (DHA, C22:6) acids. Naturally, the degree of unsaturation plays an important role in susceptibility of lipid matrices to oxidation. The relative rate of oxidation for stearic (C18:0), oleic, linoleic, and linolenic acids has been reported to be 1:100:1200:2500^17^. Arachidonic acid (C20:4) has been shown to be oxidized 2.9 times faster than linoleic acid^18^. Oxygen uptake of EPA and DHA esters after the IPs has been reported to be 5.2 and 8.5 times, respectively, faster than that of ethyl linolenate^19^.

**Table 1:** Fatty acid composition (%w/w) of the oils studied.

Fatty acid	Oil sample	
	Canola	Fish
C14:0	0.06 ± 0.02^b^	6.21 ± 0.03^a^
C16:0	4.20 ± 0.02^b^	17.31 ± 0.09^a^
C16:1	0.20 ± 0.03^b^	13.23 ± 0.00^a^
C17:0	0.10 ± 0.03^b^	1.88 ± 0.05^a^
C17:1	–	–
C18:0	2.50 ± 0.06^b^	3.21 ± 0.02^a^
C18:1	61.70 ± 0.03^a^	27.55 ± 0.15^b^
C18:2	18.70 ± 0.11^a^	8.15 ± 0.08^b^
C18:3	9.20 ± 0.37^a^	1.17 ± 0.01^b^
C20:0	0.80 ± 0.01^b^	1.15 ± 0.03^a^
C20:1	1.20 ± 0.1	–
C20:4	–	0.21 ± 0.02
C20:5 (EPA)	–	6.35 ± 0.04
C22:0	0.40 ± 0.00	–
C22:6 (DHA)	–	5.89 ± 0.06
C22:1	0.40 ± 0.02	–
C24:0	0.20 ± 0.01	–
C24:1	0.19 ± 0.03	–
SFA	8.26 ± 0.14^b^	29.78 ± 0.05^a^
MUFA	63.7 ± 0.11^a^	40.79 ± 0.14^b^
PUFA	27.90 ± 0.26^a^	21.77 ± 0.20^b^

Means ± SD (standard deviation) within a row with the same lowercase letters are not significantly different at *p* < 0.05.

*EPA* Eicosapentaenoic acid, *DHA* docosahexaenoic acid, *SFA* saturated fatty acids, *MUFA* monounsaturated fatty acids, *PUFA* polyunsaturated fatty acids.

### Kinetic data analysis

**Figure 3:**
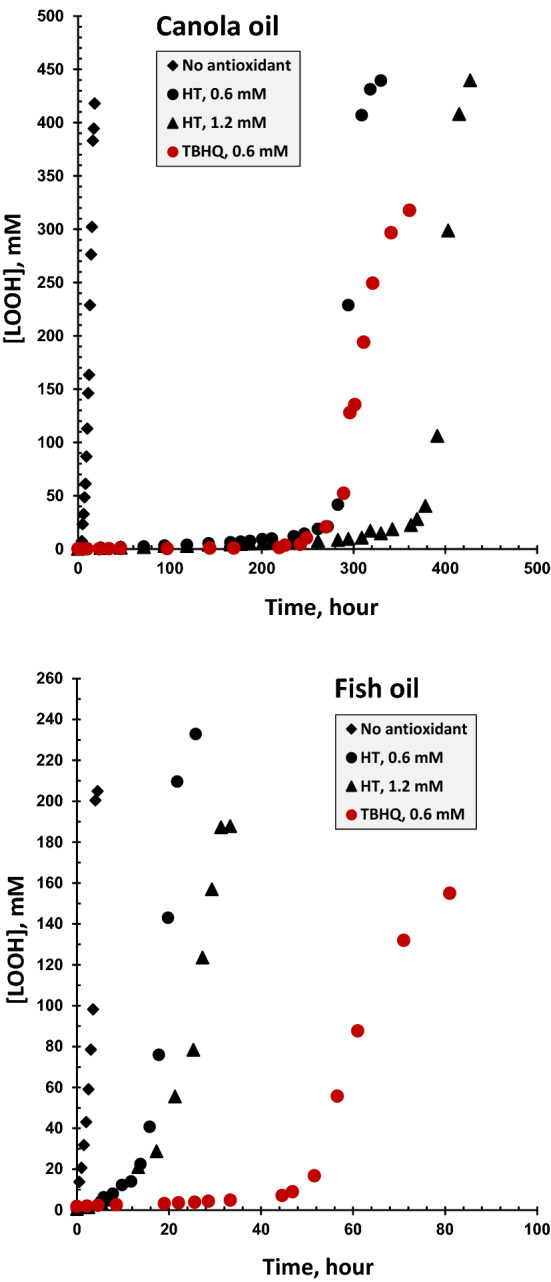
Kinetic curves of the accumulation of lipid hydroperoxides (LOOH) during the non-inhibited and inhibited (hydroxytyrosol, HT, and *tert*-butylhydroquinone, TBHQ) peroxidations of the oil samples at 60 °C.

Figure 3 illustrates the LOOH kinetic curves over the whole range of the lipid peroxidation of the stripped canola and fish oils at 60 °C. Table 2 provides a wide range of kinetic data calculated from Eqs. (2) and (3).

**Table 2:** The kinetic data resulting from the linear and sigmoidal functions fitted on the kinetic curves of the accumulation of lipid hydroperoxides (LOOH) during the non-inhibited and inhibited (hydroxytyrosol, HT, and *tert*-butylhydroquinone, TBHQ) peroxidations of the oil samples at 60 °C.

Kinetic parameter	Canola oil				Fish oil			
	No antioxidant	HT (0.6 mM)	HT (1.2 mM)	TBHQ (0.6 mM)	No antioxidant	HT (0.6 mM)	HT (1.2 mM)	TBHQ (0.6 mM)
**Initiation phase**								
IP (h)	9.32 ± 0.09^f^	284 ± 1^b^	386 ± 1^a^	280 ± 2^b^	2.53 ± 0.02^g^	16.2 ± 0.1^e^	19.3 ± 0.1^d^	50.7 ± 0.9^c^
*k*_IP_ (mM h^−1^)	4.18 ± 0.08^b^	0.0393 ± 0.0007^f^	0.0325 ± 0.0012^f^	0.0044 ± 0.0003^g^	22.3 ± 0.5^a^	1.16 ± 0.06^c^	0.6596 ± 0.0227^d^	0.0892 ± 0.0049^e^
*O*_i_ (mM^−1^ h^2^)	2.23 ± 0.05^g^	7238 ± 119^c^	11,885 ± 438^b^	63,743 ± 4546^a^	0.11 ± 0.00^h^	14.0 ± 0.7^f^	29.3 ± 1.0^e^	569 ± 27^d^
[LOOH]_IP_ (mM)	33.0 ± 2.1^b^	11.0 ± 0.3^e^	12.1 ± 0.4^d^	1.33 ± 0.07^g^	56.9 ± 1.2^a^	18.8 ± 1.1^c^	13.0 ± 0.5^d^	6.31 ± 0.21^f^
**Propagation phase**								
*t*_p_ (h)	8.05 ± 0.08^d^	19.0 ± 0.3^c^	24.6 ± 0.3^b^	50.5 ± 2.2^a^	2.14 ± 0.10^f^	6.93 ± 0.59^e^	18.8 ± 0.3^c^	18.6 ± 0.5^c^
*R*_max_ (mM h^−1^)	23.3 ± 0.2^c^	21.6 ± 0.3^d^	16.8 ± 0.4^e^	6.27 ± 0.2^h^	76.6 ± 4.2^a^	36.2 ± 2.9^b^	13.7 ± 0.2^f^	7.87 ± 0.27^g^
[LOOH]_max_ (mM)	220 ± 2^d^	422 ± 2^a^	424 ± 10^a^	318 ± 4^b^	220 ± 1^d^	268 ± 2^c^	270 ± 7^c^	152 ± 3^e^
*R*_n_ (h^−1^)	0.1056 ± 0.0014^c^	0.0512 ± 0.0008^d^	0.0395 ± 0.0004^e^	0.0197 ± 0.0009^f^	0.3479 ± 0.0192^a^	0.1349 ± 0.0116^b^	0.0506 ± 0.0007^d^	0.0517 ± 0.0013^d^
*k*_c_ (h^−1^)	0.4224 ± 0.0056^c^	0.2050 ± 0.0030^d^	0.1581 ± 0.0016^e^	0.0790 ± 0.0034^f^	1.3915 ± 0.0767^a^	0.5395 ± 0.0463^b^	0.2023 ± 0.0027^d^	0.2067 ± 0.0051^d^
*k*_d_ (mM^−1^ h^−1^)	0.001918 ± 0.000039^b^	0.000485 ± 0.000009^e^	0.000373 ± 0.000011^f^	0.000249 ± 0.000013^g^	0.006318 ± 0.000351^a^	0.002011 ± 0.000181^b^	0.000750 ± 0.000030^d^	0.001358 ± 0.000036^c^

Means ± SD (standard deviation) within a row with the same lowercase letters are not significantly different at P < 0.05.

*IP* Induction period, *k*_*IP*_ the pseudo-zero order rate constant of initiation phase, *O*_*i*_ initiation oxidizability parameter, *[LOOH]*_*IP*_ LOOH concentration at IP, called as critical reverse micelle concentration (CMC) of lipid hydroperoxides (CMC_L_), *t*_*P*_ duration of the propagation phase, *R*_*max*_ the maximum rate of LOOH accumulation, *[LOOH]*_*max*_ the maximum concentration of LOOH, *R*_*n*_ propagation oxidizability parameter, *k*_*c*_ a composite (pseudo-first order) rate constant, *k*_*d*_ the decomposition (pseudo-second order) rate constant of the bimolecular reaction of preformed LOOH.

### Initiation phase of lipid peroxidation

As expected, the canola oil was of significantly better initiation oxidizability than the fish oil (*O*_i_ = 2.23 vs. 0.11 mM^−1^ h^2^, Table 2). This was obviously due to its less highly polyunsaturated fatty acid composition (Table 1; C20:4, C20:5, and C22:6) which naturally prolongs IP and decreases *k*_IP_. Lipid systems of higher capability to generate less reactive LOO^**∙**^ show greater IPs^6^. Also, those less prone to produce free radicals of more reactivity and diversity, including LOO^**∙**^ (*E*^0^ = 1000 mV), alkoxyl (LO^**∙**^, *E*^0^ = 1600 mV), and/or hydroxyl (^**∙**^OH, *E*^0^ = 2320 mV)^20,21^, being able to initiate and propagate peroxidation^22^, provide smaller *k*_IP_ values.

The parameter *O*_i_ was extraordinarily improved in the presence of the antioxidants added (Table 2), indicating their high potency to scavenge the reactive free radicals LOO^**∙**^, LO^**∙**^, and/or ^**∙**^OH. Considering the unifying parameter *A* given in Table 3, the antioxidants acted more dramatically against the initiation peroxidation in the canola oil which possessed a reaction environment of relatively lower oxidative instability than that of the fish oil. This clearly implies the more intensive consumption of the antioxidant molecules to reduce the radicals of higher reactivity and diversity in the fish oil. Similar results have been reported to show the superior antioxidant activity of HT^9^ and TBHQ^3,8,23^ in the linoleic/linolenic acid group of edible oils.

**Table 3:** The initiation kinetics of the inhibited peroxidation of the oil samples in the presence of hydroxytyrosol (HT) and *tert*-butylhydroquinone (TBHQ) at 60 °C.

Antioxidant	*F*	ORR	*A*
**Canola oil**			
HT (0.6 mM)	30.5 ± 0.3^b^	0.0094 ± 0.0003^c^	3245 ± 108^c^
HT (1.2 mM)	41.4 ± 0.4^a^	0.0078 ± 0.0003^d^	5308 ± 211^b^
TBHQ (0.6 mM)	30.1 ± 0.4^b^	0.0011 ± 0.0001^f^	27,364 ± 2514^a^
**Fish oil**			
HT (0.6 mM)	6.40 ± 0.06^e^	0.0520 ± 0.0029^a^	123 ± 7^e^
HT (1.2 mM)	7.63 ± 0.07^d^	0.0296 ± 0.0012^b^	258 ± 11^d^
TBHQ (0.6 mM)	20.1 ± 0.4^c^	0.0040 ± 0.0002^e^	5025 ± 270^b^

Means ± SD (standard deviation) within a column with the same lowercase letters are not significantly different at P < 0.05.

*F* Stabilization factor, *ORR* oxidation rate ratio, *A* antioxidant activity.

In general, TBHQ with two hydroxyl (–OH) groups in *para* position and a tertiary butyl group [–C(CH_3_)] around the phenolic –OH group (Fig. 2) exhibited quite higher antioxidant activities than HT with two –OH groups in *ortho* position and a hydroxyethyl group (–CH_2_CH_2_OH) far from the phenolic –OH group (Fig. 2) in preventing the initiation peroxidation (Table 3). It has been postulated that the alcoholic –OH group in HT is able to orient towards the aromatic ring and to establish an intramolecular hydrogen bond with the catecholic (1,2-dihydroxybenzene) hydroxyl groups. This accordingly leads to form minimum-energy conformers which have lower tendency to reduce the oxidizing radicals^24^.

As shown in Table 2, the parameter [LOOH]_IP_ of the control samples were significantly different. According to Ghnimi et al.^25^, [LOOH]_IP_ actually represents the critical concentration of reverse micelles (CMC_L_) composed basically of the LOOH accumulated during IP. It may indicate the level of LOOH amphiphilicity and their spatially alignment in water–oil interfaces^24^. The much bigger [LOOH]_IP_ in the fish oil control can be attributed to its fatty acid composition of higher diversity and the lower amphiphilic character resulting from the lesser extent of unsaturation degree (Table 1). Diversity in fatty acid compositions arises from the length of acyl chains as well as the number of double bonds. More unsaturated fatty acids generate LOOH molecules of higher polarity and, therefore, of higher levels of surface activity, facilitating their incorporation into the water–oil interfaces^25^. However, more unsaturated LOOH would be bulkier and occupy more space in the interface, decreasing their compact aggregations^26^. The value of [LOOH]_IP_ decreased considerably when adding the antioxidants (Table 2). Phenolic antioxidants are considered as amphiphilic molecules likely to have surfactant or co-surfactant properties^25^, enabling them to decrease interfacial tension and establish more stable and organized reverse micelles. TBHQ with a hydrophobic tertiary butyl substituent showed a partition coefficient of three times higher than that of HT with a hydrophilic ethyl alcohol substituent (log P = 1.38 vs. 0.46), denoting its lesser sharing in the water–oil interfaces. Similar results were observed when evaluating the antioxidant potency of gallic acid and methyl gallate compared to TBHQ in sunflower oil triacyl glycerols^3^.

### Propagation phase of lipid peroxidation

The duration of the propagation phase (*t*_p_) is normally quite smaller than IP under mild oxidative conditions. However, they might approach together as the oxidative conditions become harsher. As can be seen in Table 2, the antioxidants could significantly change the values of *t*_p_ in both the oil samples. By analogy with the initiation kinetics of the inhibited peroxidation (Table 3), the relative quantities of *t*_p_ (*t*_p_′, Table 4) indicated more remarkable effect of HT and TBHQ on the propagation time of the fish oil possessing the fatty acid composition of naturally higher susceptibility to peroxidation (Table 1). This was in contrast to the antioxidants performance in terms of the stabilization factor *F* in the two oils (Table 3). TBHQ, in general, exerted significantly better inhibitory effects than HT on the oxidizing radicals in the propagation phase of lipid peroxidation.

**Table 4:** The relative quantities of the propagation kinetic data shown in Table 2.

HT (mM)	*t*_p_′	*R*_max_′	[LOOH]_max_′	*R*_n_′	*k*_c_′	*k*_d_′
**Canola oil**						
HT (0.6 mM)	2.36 ± 0.04^d^	0.93 ± 0.02^a^	1.92 ± 0.02^a^	0.49 ± 0.01^a^	0.49 ± 0.01^a^	0.25 ± 0.01^b^
HT (1.2 mM)	3.06 ± 0.05^c^	0.72 ± 0.02^b^	1.93 ± 0.05^a^	0.37 ± 0.01^b^	0.37 ± 0.01^b^	0.20 ± 0.01^bc^
TBHQ (0.6 mM)	6.27 ± 0.28^b^	0.27 ± 0.01^d^	1.45 ± 0.02^b^	0.19 ± 0.01^c^	0.19 ± 0.01^c^	0.13 ± 0.01^d^
**Fish oil**						
HT (0.6 mM)	3.24 ± 0.31^c^	0.47 ± 0.05^c^	1.22 ± 0.01^c^	0.39 ± 0.04^b^	0.39 ± 0.05^b^	0.32 ± 0.03^a^
HT (1.2 mM)	8.79 ± 0.43^a^	0.18 ± 0.01^e^	1.23 ± 0.03^c^	0.15 ± 0.01^d^	0.15 ± 0.01^d^	0.12 ± 0.01^d^
TBHQ (0.6 mM)	8.69 ± 0.47^a^	0.10 ± 0.01^f^	0.69 ± 0.01^d^	0.15 ± 0.01 ^d^	0.15 ± 0.01^d^	0.22 ± 0.01^b^

Means ± SD (standard deviation) within a column with the same lowercase letters are not significantly different at P < 0.05.

The maximum rate of LOOH formation during *t*_p_ (*R*_max_) was significantly affected by the antioxidants added (Table 2). This demonstrates that the antioxidants were still able to scavenge the reactive free radicals that propagate the oxidation reaction chains. Besides, the value of [LOOH]_max_ as a measure of the potency of lipid systems to create LOOH compositions of different stability^4^ significantly improved under the inhibited peroxidations. As given by Eq. (4), [LOOH]_max_ is affected by the balance between the overall formation rate of LOOH molecules (*k*_c_) and the rate of LOOH decomposition (*k*_d_)^5^, which both in turn could show well the better antioxidant performance of TBHQ than HT in the two oils (Table 2). The composite rate constant *k*_c_ has been shown to be fully correlated with the parameter *R*_n_, unifying the values of *R*_max_ and [LOOH]_max_^5^. *R*_n_ can be taken into account as a comprehensive kinetic parameter encompassing the values of every single kinetic parameter and rate constant noted above. It could significantly differentiate the antioxidant potencies in inhibiting the propagation peroxidations as affected by the type of antioxidant as well as oxidative system. With respect to the relative quantities of *R*_n_ (*R*_n_′, Table 4), HT and especially TBHQ were able to protect better the fish oil, which was more oxidizable than the canola oil, from propagation peroxidation.

## Conclusions

The present study indicated how a wide range of kinetic parameters and rate constants characterizing the initiation and propagation phases of lipid peroxidation may be changed by adding an antioxidant to the lipid systems of different degrees of unsaturation. Unlike the conventional methodology focusing on the antioxidants performance exclusively during the initiation phase of lipid peroxidation, this study demonstrated that the activity of antioxidants in the propagation phase must be taken into account as well. Interestingly, the performance of an antioxidant in the initiation and propagation phases might be quite different from each other as a function of the degree of unsaturation and the diversity in the fatty acid composition. This is of extremely high importance because the secondary oxidation products, which lead to many dramatic negative effects on sensory attributes and toxicity of lipid matrices, are likely to significantly be produced in the propagation phase of lipid peroxidation.
