# Publisher Correction: Initiation and propagation kinetics of inhibited lipid peroxidation

**DOI:** 10.1038/s41598-021-90965-2

**Published:** 2021-05-26

**Authors:** Reza Farhoosh

**Affiliations:** Department of Food Science and Technology, Ferdowsi University of Mashhad, Faculty of Agriculture, P.O. Box: 91775-1163, Mashhad, Iran

Correction to: *Scientific Reports* 10.1038/s41598-021-86341-9, published online 25 March 2021

The original version of this Article contained an error in Figure 2 where the names of the two chemical structures were interchanged. The original Figure 2 and accompanying legend appear below. 

The original Article has been corrected.

**Figure 2:**
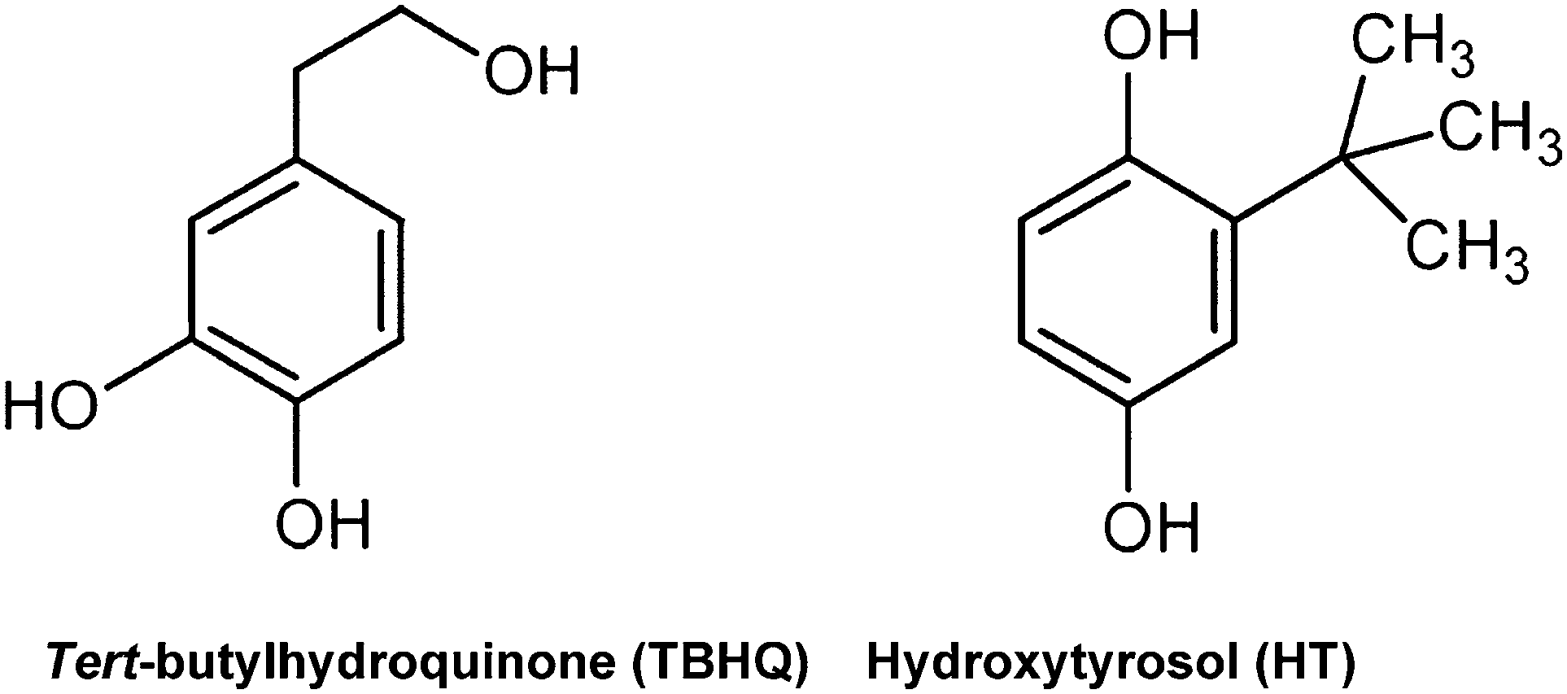
Molecular structure of hydroxytyrosol and *tert*-butylhydroquinone.

